# Skeletal Muscle Mitochondrial Protein Synthesis and Respiration Increase With Low-Load Blood Flow Restricted as Well as High-Load Resistance Training

**DOI:** 10.3389/fphys.2018.01796

**Published:** 2018-12-17

**Authors:** Thomas Groennebaek, Nichlas R. Jespersen, Jesper Emil Jakobsgaard, Peter Sieljacks, Jakob Wang, Emil Rindom, Robert V. Musci, Hans Erik Bøtker, Karyn L. Hamilton, Benjamin F. Miller, Frank V. de Paoli, Kristian Vissing

**Affiliations:** ^1^Section for Sports Science, Department of Public Health, Aarhus University, Aarhus, Denmark; ^2^Department of Cardiology, Aarhus University Hospital, Aarhus, Denmark; ^3^Department of Biomedicine, Aarhus University, Aarhus, Denmark; ^4^Department of Health and Exercise Science, Colorado State University, Fort Collins, CO, United States; ^5^Aging and Metabolism Research Program, Oklahoma Medical Research Foundation, Oklahoma City, OK, United States

**Keywords:** ischemic resistance training, deuterium oxide, bioenergetics, high-resolution respirometry, mitochondrial biogenesis

## Abstract

**Purpose:** It is well established that high-load resistance exercise (HLRE) can stimulate myofibrillar accretion. Additionally, recent studies suggest that HLRE can also stimulate mitochondrial biogenesis and respiratory function. However, in several clinical situations, the use of resistance exercise with high loading may not constitute a viable approach. Low-load blood flow restricted resistance exercise (BFRRE) has emerged as a time-effective low-load alternative to stimulate myofibrillar accretion. It is unknown if BFRRE can also stimulate mitochondrial biogenesis and respiratory function. If so, BFRRE could provide a feasible strategy to stimulate muscle metabolic health.

**Methods:** To study this, 34 healthy previously untrained individuals (24 ± 3 years) participated in BFRRE, HLRE, or non-exercise control intervention (CON) 3 times per week for 6 weeks. Skeletal muscle biopsies were collected; (1) before and after the 6-week intervention period to assess mitochondrial biogenesis and respiratory function and; (2) during recovery from single-bout exercise to assess myocellular signaling events involved in transcriptional regulation of mitochondrial biogenesis. During the 6-week intervention period, deuterium oxide (D_2_O) was continuously administered to the participants to label newly synthesized skeletal muscle mitochondrial proteins. Mitochondrial respiratory function was assessed in permeabilized muscle fibers with high-resolution respirometry. Mitochondrial content was assessed with a citrate synthase activity assay. Myocellular signaling was assessed with immunoblotting.

**Results:** Mitochondrial protein synthesis rate was higher with BFRRE (1.19%/day) and HLRE (1.15%/day) compared to CON (0.92%/day) (*P* < 0.05) but similar between exercise groups. Mitochondrial respiratory function increased to similar degree with both exercise regimens and did not change with CON. For instance, coupled respiration supported by convergent electron flow from complex I and II increased 38% with BFRRE and 24% with HLRE (*P* < 0.01). Training did not alter citrate synthase activity compared to CON. BFRRE and HLRE elicited similar myocellular signaling responses.

**Conclusion:** These results support recent findings that resistance exercise can stimulate mitochondrial biogenesis and respiratory function to support healthy skeletal muscle and whole-body metabolism. Intriquingly, BFRRE produces similar mitochondrial adaptations at a markedly lower load, which entail great clinical perspective for populations in whom exercise with high loading is untenable.

## Introduction

Aging, prolonged inactivity, and several chronic diseases negatively affect skeletal muscle mitochondrial and myofibrillar properties (Clark, 2009; Zizola and Schulze, 2013; Gram et al., 2014; Rontoyanni et al., 2017; Granata et al., 2018; Holloway et al., 2018). These negative effects may impair mobility and lead to the development of metabolic disorders such as diabetes and insulin insensitivity (Kelley et al., 2002; Schrauwen-Hinderling et al., 2007; Hesselink et al., 2016). Accordingly, strategies to improve mitochondrial and myofibrillar properties are important for skeletal muscle function and whole-body health.

Endurance-type exercise is traditionally used to stimulate mitochondrial biogenesis and to improve mitochondrial function while resistance-type exercise is traditionally used to stimulate myofibrillar accretion and strength gains (Garber et al., 2011). However, concurrent practice by traditionally recommended training principles to achieve both types of adaptations entails high exercise intensity and investment of substantial exercise time, which may be untenable in clinical populations. Interestingly, emerging evidence suggests that traditional high-load resistance exercise (HLRE) can also stimulate mitochondrial protein fractional synthesis rate (FSR) (Wilkinson et al., 2008; Donges et al., 2012). Furthermore, HLRE has been shown to improve mitochondrial respiratory function in permeabilized muscle fibers (Pesta et al., 2011; Salvadego et al., 2013; Porter et al., 2015; Holloway et al., 2018). However, it should be noted that two studies have failed to demonstrate a similar positive effect in isolated mitochondria (Irving et al., 2015; Robinson et al., 2017). This discrepancy may be attributed to disruption of mitochondrial morphology inherent to studies on isolated mitochondria (Picard et al., 2011a), which warrant further investigation into the effect of resistance exercise on mitochondrial respiratory function in permeablized fibers as well as isolated mitochondria. Collectively, these recent observations suggest that HLRE can in fact produce adaptations of importance to both myofibrillar and mitochondrial properties. Still, several clinical conditions, such as arthritis, post-surgical recovery, and advanced aging may prohibit the use of resistance exercise with high loading. Under such circumstances, blood flow restricted resistance exercise (BFRRE) could constitute a time-efficient low-load alternative. In accordance, when combined with partial restriction of blood flow, loading as low as 20–30% of maximal loading has been observed highly efficient to produce muscle hypertrophy with little time expenditure required (Fahs et al., 2015; Farup et al., 2015). However, while BFRRE is effective in stimulating muscle accretion, it is currently unknown if this approach, similar to HLRE, can also stimulate adaptations of importance to mitochondrial properties (Groennebaek and Vissing, 2017). Yet, since external restriction of blood flow during resistance exercise has been shown to augment tissue deoxygenation (Ganesan et al., 2015; Lauver et al., 2017), there is reason to believe that BFRRE could effectively stimulate such mitochondrial adaptations. In accordance ischemia is associated with perturbations in ATP turnover and reactive oxygen species (ROS) production (Korthuis et al., 1985; Kudo et al., 1995), which in turn can promote signaling for transcription of mitochondrial genes (Irrcher et al., 2009; Hood et al., 2016).

The purpose of the current study was to investigate skeletal muscle mitochondrial adaptations to 6 weeks of BFRRE and HLRE, practiced in accordance with commonly recommended guidelines (American College of Sports Medicine [ACSM], 2009; Scott et al., 2015), in young healthy subjects. Mitochondrial adaptations were assessed by using deuterium oxide (D_2_O) to assess long-term mitochondrial protein FSR as well as by measuring changes in mitochondrial content, and mitochondrial respiratory function. In addition, we measured myocellular signaling after a single bout of exercise to obtain information on the stresses driving mitochondrial adaptations to BFRRE and HLRE. We hypothesized that both BFRRE and HLRE would stimulate mitochondrial adaptations and that BFRRE would further augment such adaptations compared to HLRE.

## Materials and Methods

### Subjects

Thirtyfour young, healthy, untrained men volunteered to participate in the study. Participants were excluded from the study if they had engaged in resistance training 6 months prior to inclusion and/or if they had participated in other structured moderate/high intensity exercise training (>1 h week^-1^) 6 months prior to inclusion. Other exclusion criteria included routine strenuous work- and/or commute-related physical activity, use of prescriptive medicine with known or potential effects on muscle metabolism and growth, and intake of dietary supplements (e.g., protein and creatine supplements). Baseline subject characteristics are presented in Table 1.

**Table 1:** Baseline characteristics.

	CON	BFRRE	HLRE
n	10	12	12
Age (yr)	24 (21; 27)	23 (22; 24)	24 (23; 26)
Height (cm)	182.7 (176.9; 188.4)	180.5 (177.2; 183.7)	179.1 (176.2; 181.9)
Weight (kg)	79.1 (71.7; 86.5)	72.2 (66.3; 78.1)	82.0 (71.3; 92.7)
BMI (kg/m^2^)	23.8 (21.4; 26.1)	22.2 (20.5; 23.8)	25.54 (22.4; 28.7)


*BMI, body mass index; BFRRE, blood flow restricted resistance exercise; CON, non-exercise control; HLRE, high-load resistance exercise. Data are shown as means with 95% CI.*

Written informed consent was obtained from all participants prior to inclusion. The study was approved by the Central Denmark Region Committee on Health Research Ethics (1-10-72-218-16) and registered in the database clinicaltrials.gov (NCT03380663). The study conformed to the standards for human experimental trials outlined in the Declaration of Helsinki.

### Study Design

**FIGURE 1:**
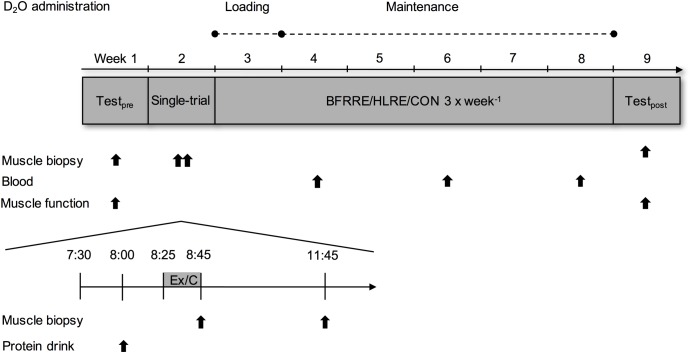
Study overview. The total study length was 9 weeks and comprised a single-trial study and a long-term study. For the single-trial study, subjects arrived in the early morning after overnight fasting. After 30 min supine rest, a whey protein supplement was administered. After consumption, participants completed a BFRRE, HLRE, or CON single-bout session. Biopsies were harvested immediately after BFRRE/HLRE/CON (45 min after protein administration) and again 3 h after BFRRE/HLRE/CON (3 h and 45 min after protein administration). During the subsequent 6-week intervention period, BFRRE and HLRE groups conducted training 3 times per week. Throughout the 6-week intervention period, D_2_O was orally administered to the participants with blood collected every second week to measure tracer enrichment. Biopsies and tests of exercise capacity were performed before and 4 days after the 6-week intervention period. BFRRE, blood flow restricted resistance exercise; CON, non-exercise control; HLRE, high-load resistance exercise; D_2_O, deuterium oxide; Ex, exercise (i.e., HLRE or BFRRE); C, non-exercise control (i.e., rest).

Participants were randomly assigned to BFRRE (*n* = 12), HLRE (*n* = 12), or a non-exercise control group (CON) (*n* = 10). For each participant, the total study length was 9 weeks and comprised participation in a single-trial study and a long-term study (Figure 1). All participants completed both the single-trial study and the long-term study.

The single-trial study was conducted to evaluate acute myocellular signaling involved in transcriptional regulation of mitochondrial adaptations. Muscle biopsies were harvested immediately (at 0 h) and 3 h after a single bout of the prescribed intervention. The long-term study was conducted to evaluate cumulative mitochondrial adaptations to 6 weeks of BFRRE and HLRE. For BFRRE and HLRE, the 6-week intervention period comprised a total of 18 exercise sessions, which has previously been demonstrated to be sufficient to promote increases in muscle functional capacity and muscle growth (Farup et al., 2015; Jakobsgaard et al., 2018). Training compliance was 100% in the BFRRE group and 98.1% in the HLRE group. 5 days before the single trial study and 4 days after cessation of the 6-week intervention period, subjects reported to the laboratory for muscle biopsies and exercise capacity testing. The CON group performed all experimental procedures except exercise.

Throughout the study period, subjects were instructed not to deviate from their habitual physical activity level. Tests of basal exercise capacity and biopsy samplings were conducted at the same absolute time points in the early morning after overnight fasting to preclude potential influence of circadian rhythm and food intake. The subjects were instructed to refrain from strenuous physical activity and alcohol 3 days prior to all tests.

### Deuterium Oxide Administration

D_2_O (99.8%, Sigma Aldrich, St. Louis, MO, United States) was orally administered to measure long-term mitochondrial protein FSR using a modified approach from our previous studies (Figure 1; Scalzo et al., 2014; Miller et al., 2015; Konopka et al., 2017). Specifically, during an initial D_2_O loading period (week 3), subjects received 2 × 1 mL per kg bodyweight during day 1 followed by 1 × 1 mL per kg bodyweight during days 2–7. During the subsequent maintenance period (weeks 4–8), subjects received 1 × 1 mL per kg bodyweight every second day. Blood samples were collected upon weeks 4, 6, and 8 to measure plasma D_2_O enrichment.

### Single-Trial Study

Prior to the 6-week intervention period, subjects participated in a single-trial study to evaluate acute myocellular signaling involved in transcriptional regulation of mitochondrial adaptations (Figure 1). Participants arrived at 7.30 am after overnight fasting. After 30 min supine rest, all subjects consumed a drink containing 20 g whey protein isolate (Whey 100 Extra Pure, Bodylab, Denmark). There is no consensus on use of feeding or fasting in studies, on acute effects but provision of a protein supplement is common in studies on protein synthesis. After consumption, participants assigned to BFRRE and HLRE completed a standardized warm-up (described in detail below) followed by a single bout of the prescribed exercise (i.e., BFRRE or HLRE – described in detail below). Participants assigned to CON performed all procedures of the single-trial study, except exercise. This was implemented to control for potential independent effects of e.g., the dietary standardization of the protocol, repeated biopsies, and/or diurnal rhythm, which has previously been justified by us to be important factors to control for (Vissing et al., 2005). Biopsies were collected from the vastus lateralis immediately (0 h) and (3 h) after completion of the single-trial study.

### Arterial Occlusion Pressure

For participants assigned to BFRRE, arterial occlusion pressure (AOP) was determined before the single-trial study for determination of individualized cuff pressures. AOP was determined in a supine position after resting for 10 min, using a Doppler (Dopplex-D900, Huntleigh Healthcare Ltd., United Kingdom) as previously described (Sieljacks et al., 2018). BFRRE was conducted with a pressure corresponding to 50% of AOP, resulting in a mean (95% CI) exercise restriction pressure of 79 mmHg (74; 84 mmHg).

### Training Intervention

One week after the single-trial study, participants initiated the 6-week intervention period. For BFRRE and HLRE groups, supervised training (see below) was conducted 3 days per week on non-consecutive days (Mondays, Wednesdays, Fridays). Before each training session, subjects completed a light standardized warm-up consisting of 5 min of low intensity cycling (∼100 W) on a stationary bicycle ergometer (Monark Ergomedic 818E, Monark, Varberg, Sweden) followed by 1 × 5 knee-extension repetitions at a load corresponding to 50% of 1 repetition maximum (RM) and 1 × 5 knee-extension repetitions at a load corresponding to 70% of 1 RM in a knee-extension apparatus (TechnoGym selection-line, TechnoGym, Italy). For participants assigned to BFRRE, a 14 cm pneumatic cuff (Delfi Medical, Vancouver, Canada) was placed around the proximal portion of the thigh and inflated to 50% of AOP utilizing a digital tourniquet (A.T.S 2200TS, Zimmer Surgical Inc., OH, United States). This relative pressure was chosen, as it minimizes discomfort while proving equally effective compared to higher relative pressures (Loenneke et al., 2015; Counts et al., 2016). With the cuffs inflated, participants performed 4 sets of isolated knee-extensions to a state of volitional fatigue with a load corresponding to 30% of 1 RM interspersed by 30 s of inter-set recovery. During inter-set recovery, cuff pressure was maintained. Participants assigned to HLRE performed 4 sets of 12 knee-extension repetitions with a load corresponding to 70% of 1RM and 3 min of inter-set recovery.

Regardless of training regimen, a contraction duty cycle of 3 s (i.e., 1.5 s durations of concentric and eccentric phases) was controlled by auditory feedback from a metronome. The load was adjusted every second week by re-testing dynamic muscle strength. Furthermore, for HLRE, the exercise load was also progressively adjusted by a 5% increase once a subject could successfully perform 12 repetitions in all 4 sets. Conversely, if 10 repetitions could not be completed in the first set of a training session, the exercise load was immediately decreased by 5% for the subsequent sets.

### Assessment of Dynamic Muscle Strength and Local Muscular Strength-Endurance Capacity

Maximal dynamic knee-extensor strength (1RM) was estimated using a 3RM-test as previously described (Brzycki, 1993). The 3RM test was conducted before the training intervention, every second week during the training intervention, and 4 days after completion of the intervention period. Local muscular strength-endurance capacity was evaluated before and 4 days after the intervention period, based on the corresponding 3RM-test. Accordingly, after completing the 3RM-test, subjects were given 10 min of rest and the load was adjusted to 30% of 1RM estimated on the corresponding visit. With a duty cycle of 3 s dictated by a metronome as described above, subjects then performed a single set of bilateral dynamic knee-extension exercise to concentric failure. The number of repetitions was used as an indication of local muscular strength-endurance capacity.

### Muscle Biopsies

Muscle biopsies were collected at 0 and 3 h after the single-trial intervention, as well as before and after the long-term intervention using the Bergström needle technique (Bergstrom, 1975) under sterile conditions and local anesthesia (1% Lidocaine, Mylon Hospital, Norway). Muscle biopsies were collected in a randomized manner with the pre, 0 h, and post-biopsies taken from one leg and the 3 h biopsy taken from the opposite leg to minimize repeated biopsy effects (Vissing et al., 2005). Importantly, all muscle biopsies were harvested from the same mid area of vastus lateralis, at the same depth in the muscle (approximately 1–2 cm), and a few centimeters distally from the distal site of occlusion.

After removal of visible fat and connective tissue, the muscle samples were allocated into separate tubes and preserved according to the respective analysis. In accordance, an aliquot of the biopsy (∼30 mg wet weight) was immediately submerged in an ice-cold relaxing buffer (BIOPS; in mmol L^-1^: 2.77 CaK_2_EGTA, 7.23 EGTA, 20 taurine, 6.56 MgCl_2_, 5.77 Na_2_ATP, 15 Na_2_phosphocreatine, 0.5 dithiothreitol and 50 4-morpholineethanesulphonic acid; pH 7.1) and transported to the laboratory to be prepared for measures of mitochondrial respiratory function. Muscle samples for immunoblotting, enzyme activity, and FSR were frozen in liquid nitrogen and stored at -80°C until further analysis.

### Tissue Preparation for Measurement of Mitochondrial Protein FSR

Mitochondrial protein FSR was determined over the 6-week intervention period from a sub-sample of the post-muscle biopsy. Body water enrichment was determined from plasma as previously described (Robinson et al., 2011; Scalzo et al., 2014; Konopka et al., 2017). To determine percentage of deuterium enriched alanine in a subcellular fraction of skeletal muscle enriched with mitochondria, we followed previously published standard operating procedures (Robinson et al., 2011; Drake et al., 2013; Scalzo et al., 2014; Konopka et al., 2017) that have been validated by both western blot and proteomic analysis of the fraction. Approximately 25–50 mg of skeletal muscle was homogenized in an isolation buffer containing 100 mM KCl, 40 mM Tris HCl, 10 mM Tris base, 5 mM MgCl2, 1 mM EDTA, and 1 mM ATP (pH 7.5), with phosphatase and protease inhibitors (HALT; Thermo Fisher Scientific, Rockford, IL, United States) with a bead homogenizer (Next Advance, Inc., Averill Park, NY, United States). After homogenization, the samples were centrifuged at 800 *g* for 10 min at 4°C. The resulting supernatant was further centrifuged at 9000 *g* for 30 min at 4°C. The resulting pellet was isolated and rinsed twice in a second isolation buffer (100 mM KCl, 10 mM Tris HCl, 10 mM Tris base, 1 mM MgCl2, 0.1 mM EDTA, 0.02 mM ATP, and 1.5% bovine serum albumin. pH 7.5) and once with distilled water. The pellet was resuspended in 250 μL of 1 M NaOH and placed on a heat block for 15 min at 50°C shaking at 900 rpm. The mitochondrial protein enriched fraction was then incubated in 6 N HCl for 24 h at 120°C for protein hydrolysis. The hydrolysates were ion exchanged, dried in a vacuum, and resuspended in 1 mL of molecular biology grade H_2_O. Half of the suspended sample was derivatized by a 1 h incubation of 500 μL acetonitrile, 50 μL K_2_HPO_4_, pH 11, and 20 μL of pentafluorobenzyl bromide. Ethyl acetate was added and the organic layer was removed, dried under nitrogen gas, and reconstituted in 600 μL ethyl acetate for analysis on an Agilent 7890A GC coupled to an Agilent 5975C MS as previously described (Robinson et al., 2011; Scalzo et al., 2014; Konopka et al., 2017). The newly synthesized fraction (*f*) of mitochondrial proteins was calculated from the enrichment of alanine bound in muscle proteins over the entire labeling period, divided by the true precursor enrichment (*p*), using the average plasma D_2_O enrichment over the period of measurement with MIDA adjustment (Busch et al., 2006).

### Preparation of Permeabilized Muscle Fibers

The muscle sample was carefully dissected into two separated muscle fiber bundles (∼2.5 mg wet weight) in ice-cold BIOPS using the tip of two sharp forceps. The remaining muscle tissue was immediately frozen in liquid nitrogen for later analysis of citrate synthase activity. The muscle fiber bundles were chemically permeabilized by gentle agitation for 30 min in ice-cold BIOPS buffer containing saponin (50 μg mL^-1^) as previously described (Anderson et al., 2009). Following permeabilization, the fiber bundles were washed twice by gentle agitation for 10 min in an ice-cold respiration medium (MiR05; in mmol L^-1^: 110 sucrose, 60 K-lactobionate, 0.5 EGTA, 0.1% BSA, 3 MgCl_2_, 20 taurine, 10 KH_2_PO_4_ and 20 HEPES; pH 7.1). Finally, fiber bundles were blotted dry and weighed on a microbalance (Mettler-Toledo, Greifensee, Switzerland).

### High Resolution Respirometry

Mitochondrial respiratory function was measured in permeabilized muscle fibers by high-resolution respirometry using an Oxygraph-2k (Oroboros Instruments, Innsbruck, Austria). Prior to each experiment, the polarographic oxygen sensors were calibrated in 2 mL MiR05. All experiments were conducted in duplicate at 37°C and in a hyperoxygenated environment to preclude potential O_2_ diffusion limitations. We used a modified substrate/uncoupler/inhibitor-titration protocol from a previous study, designed to investigate various functional characteristics of the mitochondria (Jespersen et al., 2017). Accordingly, state 2 leak respiration supported by electron flow from complex I (GM) was measured by titration of glutamate (10 mmol L^-1^) and malate (2 mmol L^-1^). Subsequently, ADP (5 mmol L^-1^) was added to yield information on coupled state 3 respiration supported by electron transfer from complex I (GM3). Integrity of the outer mitochondrial membrane was tested by cytochrome c (10 μmol L^-1^) with an increase in respiration of >10% considered as a sign of damage leading to exclusion of data. Maximal coupled state 3 respiration supported by electron transfer from complex I and II (GMS3) was assessed by addition of succinate (10 mmol L^-1^). State 4 respiration (4o) was then analyzed by addition of oligomycin (2 μg mL^-1^). Following titration of oligomycin, maximal uncoupled respiration (E) was measured by stepwise titration of FCCP (0.5 μmol L^-1^). Finally, residual oxygen consumption (ROX) was measured by addition of rotenone (0.5 μmol L^-1^) and antimycin A (2.5 mmol L^-1^). Individual titrations were performed with a minimum of 5 min intervals. If the signal was not stable, an additional 5 min was given. Inhibitor titrations (oligomycin, rotenone and antimycin A) were performed with a minimum of 10 min to allow stable inhibition. Steady state respiratory rates were evaluated as average JO2 (oxygen consumption) over the stable period of the respiratory state using Datlab 6 software. Steady state respiratory rates (pmol s^-1^) are expressed relative to milligram wet weight of muscle tissue. Data analysis was performed by a blinded investigator.

### Citrate Synthase Activity

Frozen muscle tissue (∼25 mg wet weight) was freeze-dried for 24 h using a freeze-dryer (Alpha 1-2 LDplus, Martin Christ Gefriertrocknungsanlagen GmbH, Germany) and homogenized for 2 min in 1.3 mL 0.3 mol L^-1^ K_2_HPO_4_ with 0.05% BSA/2 mg tissue using a tissuelyser II (Qiagen, Venlo, Netherlands). Fifteen minutes after addition of 10% Triton X-100 (10 μL/mL), insoluble materials were removed by centrifugation at 10,000 ×*g* for 10 min at 4°C, and the supernatant stored for analysis of citrate synthase activity and total protein concentration. Total protein concentration was measured using a pierce 660 nm protein assay (Cat. 22660, Thermo Fisher Scientific, Rockford, IL, United States). Citrate synthase activity was measured spectrophotometrically using a citrate synthase activity assay kit (Cat. CS0720, Sigma-Aldrich, St. Louis, MO, United States). Briefly, homogenates were diluted 25 times in a reaction mix (Assay Buffer, 30 mM Acetyl CoA solution, 10 mM DTNB solution) and loaded on a 96 well half plate. After measuring background activity at wavelength 412 nm, 5 μL oxaloacetate (10 mmol L^-1^) was added to each well to initiate the reaction. Light absorbance at 412 nm was measured every 10 s for 5 min at 37°C using a spectrophotometer (PHERAstar FS, BMG LABTECH, Ortenberg, Germany). Citrate synthase activity was calculated from the linear change in absorbance over time and normalized to total protein concentration.

### Immunoblotting

After homogenization of freeze-dried muscle tissue, equal amounts of protein were resolved by sodium dodecyl sulfate poly-acrylamide gel electrophoresis (SDS-PAGE) and electroblotted onto polyvinylidene difluoride (PVDF) membranes as previously described (Rahbek et al., 2015). Protein concentration was assessed with a Bradford Assay (BioRad, CA, United States). Membranes were blocked for 2 h in 0.1% I-block (Applied Biosystems, CA, United States) TBST solution followed by overnight incubation with primary antibodies. All primary antibodies were purchased from Cell Signaling Technology and utilized as follows; p-ACC^Ser79^ (cat #3661, conc. 1:1000 in 5% BSA), p-AMPK^Thr172^ (cat #2531, conc. 1:1000 in 5% BSA), p-CaMKII^Thr286^ (cat #12716, conc. 1:1000 in 5% BSA) p-CREB^Ser133^ (cat #9198, conc. 1:1000 in 5% BSA), p-p38 MAPK^Thr180/Tyr182^ (cat #4511, 1:1000 in 5% BSA), p-p53^Ser15^ (cat #9286, 1:1000 in 5% skim milk). Subsequently, membranes were incubated with secondary antibodies for 1 h with horseradish peroxidase-conjugated goat anti-rabbit (cat #6721 ABCAM, Cambridge, United Kingdom), except for p-p53, which was incubated with horseradish peroxidase-conjugated goat anti-mouse (cat #2055, Santa Cruz, TX, United States). A concentration of 1:5000 in 1% BSA was used for all secondary antibody solutions, except for p-AMPK and p-CaMKII antibody solutions where a concentration of 1:3000 in 1% BSA was used. Proteins were visualized by chemiluminescence (Thermo Fisher Scientific, MA, United States) and quantified with an UVP imaging system (UVP, CA, United States). Phospho-specific arbitrary protein intensity was normalized to total protein (i.e., total amount of protein loaded in the corresponding lanes) using Stain Free Technology as previously described (Gurtler et al., 2013). Results are presented as fold changes from pre.

### Statistical Analyses

A one-way ANOVA was used to determine differences between groups in mitochondrial protein FSR. To compare total training volume and average time under ischemia per training session between BFRRE and HLRE, unpaired *t*-tests were utilized. Local muscular strength-endurance capacity, dynamic muscle strength, mitochondrial respiratory function, citrate synthase activity, and immunoblotting data, were analyzed by use of a linear mixed model with group, time, and time × group interaction as the factors of interest. Model validation included tests for equal standard deviations and examination of QQ-plots. We performed Pearson’s correlation analysis on mitochondrial protein FSR and CS activity, and on mitochondrial protein FSR and measures of mitochondrial function with all groups pooled, the exercise groups pooled, and individual exercise groups. All statistical analyses were performed using STATA 15.0 (StataCorp, College Station, TX, United States) with *P* < 0.05 considered a statistically significant outcome. Graphic data are presented as means ± SD, whereas in text and in table data are presented as means with 95% CI.

## Results

### Training Progression

Mean (95% CI) number of knee-extension repetitions performed in the first, second, third, and fourth set by BFRRE was 35.5 (31.8; 38.3), 11.6 (10.0; 13.2), 9.1 (7.9; 10.4), 8.4 (7.2; 9.5); by HLRE 11.4 (10.9; 11.8), 10.6 (9.9; 11.2), 9.6 (8.1; 10.42), 8.8 (8.1; 9.5). Average total volume of work (rep × load) performed was 59.7% higher for HLRE [57776.0 kg (49597.9; 65954.2 kg)] compared to BFRRE [36182.5 kg (31557.7; 40807.3 kg)] (*P* < 0.01). Average time under ischemia per training session (calculated as time under tension for HLRE and time under tension + inter-set recovery for BFRRE) was 135.8% longer for BFRRE [285.6 s (269.2; 302.0 s)] compared to HLRE [121.1 s (113.6; 128.6 s)] (*P* < 0.01).

### Muscle Functional Capacity

**FIGURE 2:**
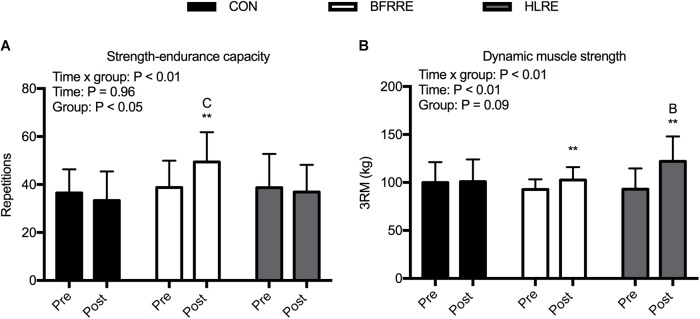
Muscle functional capacity for CON, BFRRE, and HLRE groups. **(A)** strength-endurance capacity. **(B)** Dynamic muscle strength. ^∗∗^ denotes difference from pre-values within groups (*P* < 0.01), C denotes difference from CON at corresponding time point (*P* < 0.05), B denotes difference from BFRRE at corresponding time point (*P* < 0.05). Data are presented as means ± SD. Overall effects are designated in the upper left corner of each graph.

There was an overall time × group interaction for local muscular strength-endurance capacity (*P* < 0.01) (Figure 2A). BFRRE increased strength-endurance capacity by an average of 28.3% (5.5; 51.0%) from pre to post-intervention (*P* < 0.01). There were no changes in strength-endurance capacity for HLRE and CON. There was a difference in strength-endurance capacity between CON and BFRRE post-intervention (*P* < 0.05). There were no differences in strength-endurance capacity between BFRRE and HLRE.

There was an overall time × group interaction for dynamic muscle strength (*P* < 0.01) (Figure 2B). BFRRE increased dynamic muscle strength by an average of 10.7% (4.4; 17.0%) from pre to post-intervention (*P* < 0.01). HLRE increased dynamic muscle strength by an average of 33.0% (17.5; 48.5%) from pre to post-intervention (*P* < 0.01). There were no changes in dynamic muscle strength for CON. There was a difference in dynamic muscle strength between BFRRE and HLRE post-intervention (*P* < 0.05).

### Mitochondrial Protein Synthesis

**FIGURE 3:**
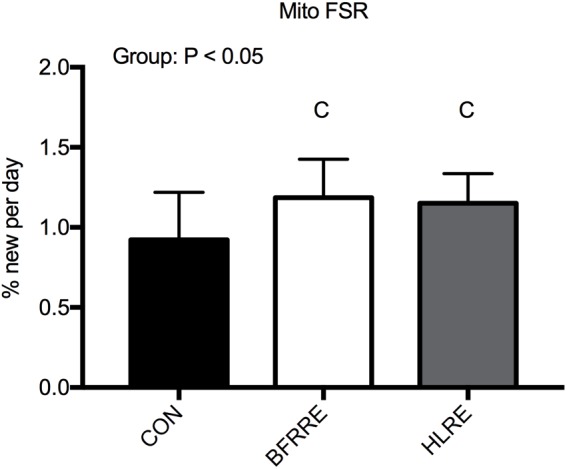
Mitochondrial protein fractional synthesis rate (mito FSR) for CON, BFRRE, and HLRE groups. C denotes difference from CON (*P* < 0.05). Data are presented as means ± SD. Overall effect is designated in the upper left corner of the graph.

Average body water enrichment remained stable through the labeling period [week 4: 0.80% (0.72; 0.87%)], week 6: 0.89% (0.79; 0.99%), week 8: 0.89% (0.76; 1.01%). Both resistance exercise training regimens increased (*P* < 0.05) mitochondrial protein FSR (%/day) compared to CON (Figure 3) [CON: 0.92%/day (0.71; 1.13%/day), HLRE: 1.15%/day (1.03; 1.27%/day), and BFRRE: 1.19%/day (1.02; 1.35%/day)]. There was no difference between BFRRE and HLRE.

### Citrate Synthase Activity

**FIGURE 4:**
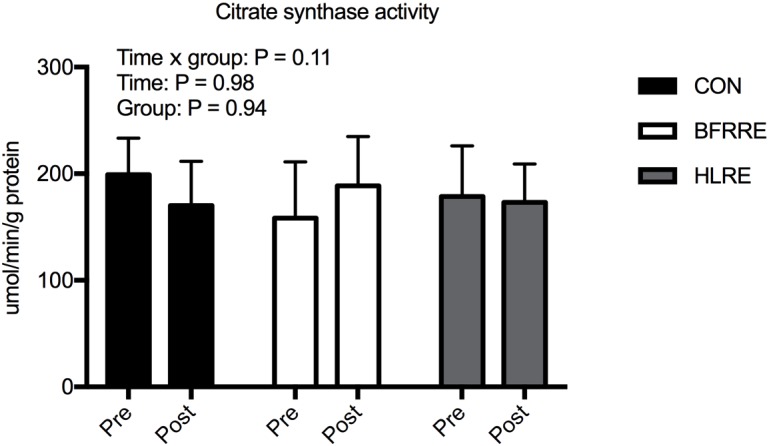
Citrate synthase activity normalized to total protein for CON, BFRRE, and HLRE groups. Data are presented as means ± SD. Overall effects are designated in the upper left corner of the graph.

For citrate synthase activity, there were no overall time × group interaction, time, or group effects (Figure 4).

### Mitochondrial Respiratory Function

**FIGURE 5:**
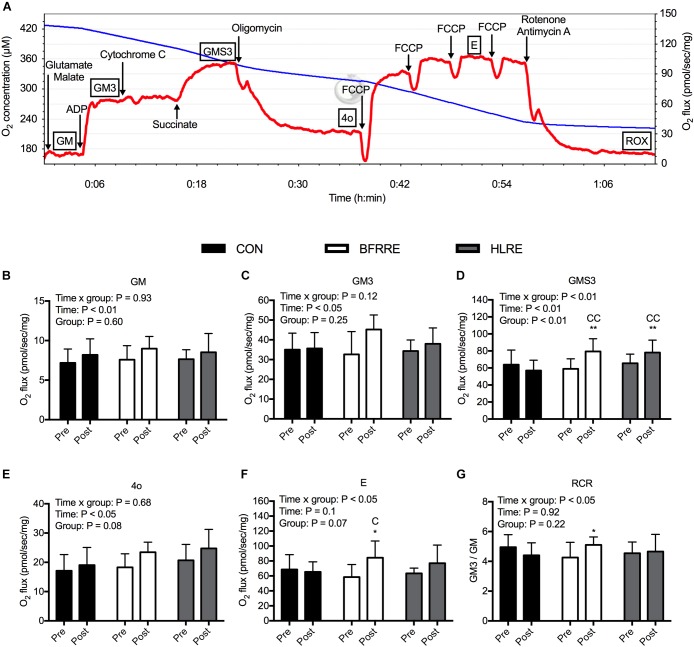
Mitochondrial respiratory function in permeabilized muscle fibers for CON, BFRRE, and HLRE groups. **(A)** representative real-time oxygraph readout showing oxygen concentration in the chamber (blue line) and the calculated negative time derivative (oxygen flux) normalized to mg wet weight of muscle tissue (red line). Titrations are denoted with arrows and respiratory states are denoted with boxes. **(B)** state 2 respiration with glutamate and malate (GM). **(C)** state 3 respiration supported electron flow from complex I (GM3). **(D)** state 3 respiration supported by electron flow from complex I and II (GMS3). **(E)** state 4 respiration with oligomycin (4o). **(F)** maximal uncoupled respiration with FCCP (E). **(G)** respiratory control ratio (RCR) with complex I linked substrates. ^∗^ denotes difference from pre-values within groups (*P* < 0.05), ^∗∗^ denotes difference from pre-values within groups (*P* < 0.01), C denotes difference from CON at corresponding time point (*P* < 0.05), CC denotes difference from CON at corresponding time point (*P* < 0.01). Data are presented as means ± SD. Overall effects are designated in the upper left corner of each graph.

Steady state respiratory rates per mg wet weight of muscle tissue are shown in Figure 5, with a representative graph of real-time oxygraph traces shown in Figure 5A. For state 2 leak respiration supported by electron flow from complex I (GM, Figure 5B), there was an overall effect of time (*P* < 0.01). Similarly, for state 3 respiration supported by electron flow from complex I (GM3, Figure 5C), there was an overall effect of time (*P* < 0.05), but the change over time was not statistically different between groups (*P* = 0.12). For state 3 respiration supported by convergent electron flow from complex I and II (GMS3, Figure 5D), there was an overall time × group interaction (*P* < 0.01). GMS3 increased by 37.6% (11.7; 63.5%) with BFRRE (*P* < 0.01) and 24.0% (7.1; 40.9%) with HLRE (*P* < 0.01). There were no changes in GMS3 for CON. After the intervention, GMS3 was higher with BFRRE (*P* < 0.01) and HLRE (*P* < 0.01) compared to CON. There were no differences in GMS3 between BFRRE and HLRE. For state 4 respiration with oligomycin (4o, Figure 5E), there was an overall effect of time (*P* < 0.05). We observed an overall time × group interaction for maximal uncoupled respiration (E, Figure 5F) (*P* < 0.05). E increased by 69.5% (18.6; 120.5%) with BFRRE (*P* < 0.05). There were no changes in E for CON and HLRE. After the intervention, E was higher with BFRRE (*P* < 0.05) compared to CON. There were no differences in E between BFRRE and HLRE. We observed an overall time × group interaction for the respiratory control ratio (RCR, Figure 5G) (*P* < 0.05). RCR increased by 19.8% (-5.7; 45.3%) with BFRRE (*P* < 0.05). There were no changes in RCR for CON and HLRE. There were no differences in RCR between BFRRE and HLRE.

In average, the fiber bundles weighed 2.48 mg (2.33; 2.62 mg). Six fiber bundles were excluded from data analysis based on the cytochrome c test. In these cases, data stem from one fiber bundle. In the cytochrome c test negative fiber bundles, the addition of cytochrome c led to an increase in respiration of 0.36% (-0.73; 1.45%) confirming the integrity of the outer mitochondrial membrane.

### Association Between Mitochondrial Protein FSR and Measures of Mitochondrial Function and Content

The correlation analyses are shown in Supplementary Table 1. Mitochondrial FSR was negatively correlated to the change in GMS3 specifically in the HLRE group (*P* < 0.05). Mitochondrial FSR was positively correlated to post-intervention GM3 and GMS3 when all groups were pooled (*P* < 0.05).

### Acute Myocellular Signaling

**FIGURE 6:**
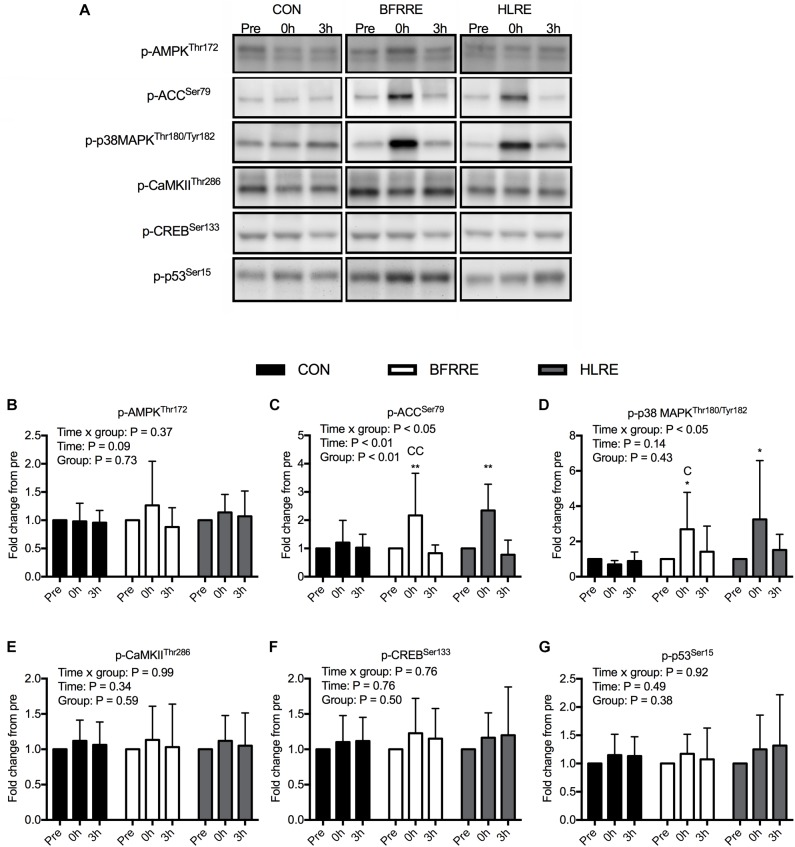
Phosphorylation levels of signaling proteins involved in regulation of mitochondrial adaptations for CON, BFRRE, and HLRE groups. **(A)** representative immunoblots for all proteins at all conditions. **(B)** phosphorylated 5′ AMP-activated protein kinase (p-AMPK). **(C)** phosphorylated acetyl-CoA carboxylase (p-ACC). **(D)** phosphorylated p38 mitogen-activated protein kinase (p-p38 MAPK). **(E)** phosphorylated calcium/calmodulin-dependent protein kinase II (p-CaMKII). **(F)** phosphorylated cAMP response-element binding protein (p-CREB). **(G)** phosphorylated p53 (p-p53). ^∗^ denotes difference from pre-values within groups (*P* < 0.05), ^∗∗^ denotes difference from pre-values within groups (*P* < 0.01), C denotes difference from CON at corresponding time point (*P* < 0.05), CC denotes difference from CON at corresponding time point (*P* < 0.01). Data are presented as mean fold changes ± SD. Overall effects are designated in the upper left corner of each graph.

Phosphorylation levels of signaling proteins involved in transcriptional regulation of mitochondrial adaptations and long-chain fatty acid uptake in the mitochondria are shown in Figure 6 with representative immunoblots shown in Figure 6A. We observed an overall time × group interaction for p-p38 MAPK (*P* < 0.05) (Figure 6D). Phosphorylation of p38 MAPK increased 2.69-fold (1.36; 4.02-fold) (*P* < 0.05) and 3.25-fold (1.13; 5.37-fold) (*P* < 0.05) at 0 h with BFRRE and HLRE, respectively. There were no changes in p-p38 MAPK for CON. There was a difference in p-p38 MAPK between CON and BFRRE at 0 h (*P* < 0.05). There were no differences in p-p38 MAPK between BFRRE and HLRE. We observed an overall time × group interaction for p-ACC (*P* < 0.05) (Figure 6C). Phosphorylation of ACC increased by 2.17-fold (1.22; 3.12-fold) (*P* < 0.01) and 2.34-fold (1.75; 2.93-fold) (*P* < 0.01) at 0 h with BFRRE and HLRE, respectively. There were no changes in p-ACC for CON. There was a difference in p-ACC between CON and BFRRE at 0 h (*P* < 0.01). There were no differences in p-ACC between BFRRE and HLRE. For p-AMPK (Figure 6B), p-CaMKII (Figure 6E), p-CREB (Figure 6F), and p-p53 (Figure 6G), there were no overall time × group interaction, time, or group effects (for specific *P*-values see respective figures).

## Discussion

The present study is the first to investigate if low-load BFRRE stimulates skeletal muscle mitochondrial adaptations. We trained healthy young individuals by commonly recommended principles of practice of BFRRE or HLRE (American College of Sports Medicine [ACSM], 2009; Scott et al., 2015). By this approach, we show that BFRRE can stimulate long-term mitochondrial protein FSR and mitochondrial respiratory function in a manner similar to HLRE. These results support recent findings that resistance-type exercise can stimulate mitochondrial adaptations to support healthy skeletal muscle and whole-body metabolism (Wilkinson et al., 2008; Pesta et al., 2011; Donges et al., 2012; Porter et al., 2015; Holloway et al., 2018). Interestingly, BFRRE achieves similar mitochondrial adaptations at a markedly lower mechanical load, which have important implications in clinical situations that prohibit high loading, e.g., under post-surgery conditions, in patients suffering from arthritis, or in elderly frail individuals.

### Mitochondrial Adaptations to Prolonged Resistance Training

In the present study, we used D_2_O to assess long-term mitochondrial protein FSR. Both BFRRE and HLRE increased mitochondrial protein FSR, demonstrating that both exercise regimens stimulate long-term increases in mitochondrial biogenesis (Miller and Hamilton, 2012). Two previous studies used short-term primed continuous amino acid infusions to demonstrate acute increases in mitochondrial protein FSR in the recovery from unaccustomed single-trial HLRE (Wilkinson et al., 2008; Donges et al., 2012). In the study by Wilkinson et al. (2008) stimulation of mitochondrial protein FSR was attenuated when a similar single bout of HLRE was performed after 10 weeks of training habituation. In another study by Robinson et al., utilizing short-term primed continuous amino acid infusions, resting mitochondrial protein FSR was measured after overnight fast before and after a 12-week HLRE intervention (Robinson et al., 2017). In this study, HLRE significantly increased resting mitochondrial protein FSR in healthy older individuals but not in the young individuals. Our study extends these previous findings by measuring cumulative mitochondrial protein FSR by use of D2O, as previously accounted for (Simmons et al., 2016; Wilkinson et al., 2017). Most importantly, this approach allowed us to assess the practical benefits of the training protocols under free-living conditions so that all components of a 6-week training period (activity changes, changes in feeding status and diuernal rythm) were considered. By this approach, we show that both HLRE and BFRRE can increase cumulative mitochondrial protein FSR. However, we acknowledge that we cannot decipher the extent to which the synthetic response was primarily driven by large increases in the early phase of the training period, so this aspect warrants further investigation.

Both BFRRE and HLRE increased maximal coupled respiration supported by convergent electron flow from complex I and II (GMS3), which is suggestive of increased mitochondrial ATP-production capacity. This is in agreement with previous findings that HLRE can promote coupled respiration in permeabilized muscle fibers (Salvadego et al., 2013; Porter et al., 2015; Holloway et al., 2018). Two other studies have failed to demonstrate a similar effect of HLRE, but these studies measured respiration in isolated mitochondria (Irving et al., 2015; Robinson et al., 2017). The differences between studies can be attributed to disruption of mitochondrial morphology and organelle interactions inherent to studies on isolated mitochondria (Picard et al., 2011a,b).

Despite that BFRRE and HLRE increased mitochondrial biogenesis and mitochondrial respiratory function (GMS3), citrate synthase activity, a commonly used marker of mitochondrial content, did not change. While this is the first study to measure the effect of BFRRE on markers of mitochondrial content most previous studies have shown no changes or even decreases in response to HLRE (Groennebaek and Vissing, 2017). As previously discussed, it is not surprising that changes in mitochondrial biogenesis are not mirrored by similar changes in mitochondrial content (Miller and Hamilton, 2012; Miller et al., 2015). Increased mitochondrial biogenesis with maintained mitochondrial content suggests that mitochondrial breakdown also increased to increase overall protein turnover. The combination of increased mitochondrial biogenesis (assessed by mitochondrial protein synthesis), maintained mitochondrial content (assessed by citrate synthase) and increased rates of mitochondrial oxygen consumption (assessed by respirometry), provides strong evidence that BFRRE and HLRE stimulate mitochondrial remodeling to improve mitochondrial function (Drake et al., 2016; Ryu et al., 2016). However, we acknowledge that we do not provide direct evidence on activation of mitochondrial breakdown. Furthermore, we emphasize that while citrate synthase activity correlates with electron microscopy-derived measures of mitochondrial content (i.e., mitochondrial volume density) (Larsen et al., 2012; Meinild Lundby et al., 2017), exercise induced changes in citrate synthase activity and mitochondrial volume density do not always correlate well (Meinild Lundby et al., 2017; Granata et al., 2018). In accordance, the extent to which differentiated resistance exercise regimens facilitate changes in mitochondrial content deserves further attention with the use of more rigorous techniques (e.g., electron microscopy).

Neither resistance training regimen improved coupled respiration supported solely by complex I substrates (GM3). This is in contrast to observations from a study on the effects of HLRE by Porter et al. (2015). This discrepancy may relate to different durations of the training interventions (6 weeks vs. 12 weeks), different exercise modes (single-limb vs. whole-body exercise), or a slightly different titration protocol. Interestingly, BFRRE increased the respiratory control ratio (RCR) with complex I substrates, calculated as the ratio between GM3 and GM, which suggests a tighter coupling between electron transfer and phosphorylation. No such effect was observed with HLRE, which is in accordance with Porter et al. (2015). On the other hand, Salvadego et al. (2013) observed a higher RCR in highly resistance trained subjects compared to sedentary controls (Salvadego et al., 2013). In the present study, BFRRE increased maximal uncoupled respiration (E), while HLRE did not. Porter et al. (2015) observed an increase in maximal uncoupled respiration in response to HLRE, but again, this discrepancy may be attributed to different training interventions and/or titration protocols. In summary, the functional mitochondrial adaptations to BFRRE at minimum match those of HLRE.

BFRRE training increased strength-endurance capacity when assessed at the same relative load while HLRE did not. HLRE training could have improved strength-endurance capacity had the test been conducted at the same absolute load. Since the chronic mitochondrial adaptations were similar between the two resistance exercise regimes, it is not clear if improvements in mitochondrial function and oxygen utilization mediated the differentiated adaptations in strength-endurance capacity with BFRRE and HLRE. It is possible that local oxygen consumption and/or capillary density differed, and that this may have contributed to differentiated adaptations in strength-endurance capacity with BFRRE and HLRE (Robbins et al., 2011), but for practical reasons we did not measure leg VO_2_ and capillary density. Another likely explanation is that the BFRRE and HLRE have different neural innervation patterns. In accordance, BFRRE likely recruits smaller motor units until onset of fatigue development with accumulating work, whereas HLRE primarily recruits larger motor units throughout the work (Henneman, 1957; Farup et al., 2015). In contrast to strength-endurance capacity, both exercise regimens improved dynamic muscle strength. However, HLRE produced a more pronounced increase in dynamic muscle strength compared to BFRRE, which further support the concept of different neural innervation patterns.

### Myocellular Signaling Underlying Mitochondrial Adaptations

In general, signaling responses to an acute bout of exercise were similar with both resistance exercise regimens.

It is known that acute perturbations in ATP turnover, calcium homeostasis, and/or ROS production stimulate mitochondrial adaptations (Hood et al., 2016). To obtain information on the type of stresses driving mitochondrial adaptations to BFRRE, we looked at responses to an acute exercise bout prior to the long-term training study. To account for ATP consumption and energy disruption associated with muscle contractions (Kjobsted et al., 2018) and/or ischemia (Kudo et al., 1995) we assessed AMPK activation through quantification of p-AMPK. We found that p-AMPK did not increase compared to CON with either training regimen. Previous studies measuring p-AMPK in response to resistance exercise have reported divergent results (Coffey et al., 2006; Wilkinson et al., 2008; Camera et al., 2010; Vissing et al., 2013). Discordance between these studies may relate to differences in dietary premise or timing of muscle biopsy sampling. In the current single-trial study, subjects were given a whey protein supplement to examine signaling changes under the common dietary approach of protein supplementation. It is possible that insulin and/or leucine may have promoted Akt-mediated phosphorylation of AMPKα Ser485/491 to antagonize phosphorylation of AMPKα Thr172 (Horman et al., 2006; Valentine et al., 2014; Kido et al., 2017). On the other hand, p-ACC, which is a downstream target of AMPK involved in signaling for fatty acid uptake into the mitochondria (McGee and Hargreaves, 2010), increased with both training regimens. p-ACC may therefore provide a read-out for AMPK activation (Richter and Ruderman, 2009), although other factors are also able to exert regulation on ACC (Brownsey et al., 2006). To control for independent effects of diet, diurnal rhythm, and repeated biopsies, we implemented a separate non-exercise control group. Since no changes occurred with CON, this indicates that metabolic perturbations imposed by resistance exercise can engage in signaling for regulation of long-chain fatty acid entry into the mitochondria (Abu-Elheiga et al., 2001). However, we do acknowledge that we did not accustom the participants to the exercise stimuli prior to the single-trial study. Consequently, signaling responses may not be representative of an exercise-familiarized state but may reflect an overall stress response typical of unaccustomed exercise (Benziane et al., 2008; Wilkinson et al., 2008). The signaling results should be interpreted with this in mind.

As for the ability of AMPK and CaMKII to engage in transcriptional regulation for mitochondrial adaptations, the observed lack of change in phosphorylation of AMPK and CaMKII suggests that the magnitude of perturbations in ATP turnover and calcium homeostasis were too modest to stimulate this (Chin, 2005; Kjobsted et al., 2018). This is supported by the simultaneous lack of phosphorylation changes in downstream transcription factors CREB and p53, both assumed to be involved in transcription of genes encoding mitochondrial proteins (Valsecchi et al., 2013; Bartlett et al., 2014). Opositely, p-p38 MAPK increased with both resistance exercise regimens. Increased phosphorylation of p38 MAPK could be ascribed to increased ROS production (Zhang et al., 2014), as result of occlusion-reperfusion during contraction and/or blood flow restriction, or to mechanical stress *per se* (Zhan et al., 2007).

In the current study, signaling responses for metabolic adaptations were similar between BFRRE and HLRE. In a previous study, it has been shown that acute low-intensity cycling with blood flow restriction has little effect on metabolic signaling compared to traditional resistance training and endurance training (Smiles et al., 2017). Furthermore, it has been shown that application of external restriction of blood flow after sprint-interval training does not further accentuate phosphorylation of p-38 MAPK (Taylor et al., 2016). The effect of differentiated blood-flow restricted exercise regimens on signaling for metabolic adaptations deserves further attention with experimental designs that allow for more direct comparison.

### Clinical Perspectives

The primary aim of our study was to determine if BFRRE could effectively promote mitochondrial adaptations. The rationale for this investigation was that low-load BFRRE imposes less force on the joints and may provide a more feasible alternative to HLRE for those recovering from surgery, in patients suffering from arthritis or in elderly frail individuals (Hughes et al., 2017). We have previously demonstrated that 6 weeks of low-load BFRRE and low-load free-flow resistance exercise performed to volitional fatigue were equally capable of producing pronounced muscle hypertrophy but with much less work and time expenditure per session required with BFRRE (Farup et al., 2015). In this context, the current findings are exciting. With regard to feasibility, it can be argued that even though BFRRE performed to fatigue is time-efficient, it can be experienced as uncomfortable in the unfamiliar state. However, in a previous study, we provided evidence that repeated bouts attenuate sensation of discomfort, similarly, to other types of unfamiliar exercise (Sieljacks et al., 2016). Moreover, all the subjects of the current study completed all training sessions without experiencing adverse events. Still, we acknowledge that similar studies need to be conducted in patient populations, to ultimately prove feasible and recommendable in the clinic.

## Conclusion

The present study demonstrates that prolonged BFRRE as well as HLRE can stimulate long-term mitochondrial protein synthesis and increase mitochondrial respiratory function. Our results support that resistance exercise can promote important muscle metabolic adaptations and that the use of a low-load resistance exercise regimen is equally effective for achieving such adaptations. Future studies should focus on the use of BFRRE as an alternative to HLRE in clinical situations that prohibit high loading.

## Author’s Note

The study was conducted at Section for Sports Science, Department of Public Health, Aarhus University.

## Author Contributions

TG, PS, ER, KH, BM, HB, FdP, and KV contributed to the conception and design. TG and KV wrote the first manuscript draft. All authors contributed to data acquisition and/or interpretation of data, critically revised the manuscript and provided intellectual contributions, and approved the final version of the manuscript submitted for publication.

## Conflict of Interest Statement

The authors declare that the research was conducted in the absence of any commercial or financial relationships that could be construed as a potential conflict of interest.

## Abbreviations

4o: state 4 respiration with oligomycin
ACC: acetyl-CoA carboxylase
AMP: adenosine monophosphate
AMPK: 5′ AMP-activated protein kinase
ANOVA: analysis of variance
AOP: arterial occlusion pressure
ATP: adenosine triphosphate
BFRRE: blood flow restricted resistance exercise
CaMKII: calcium/calmodulin dependent protein kinase II
CI: confidence interval
CON: non-exercise control
CREB: cAMP response-element binding protein
D_2_O: deuterium oxide
E: maximal uncoupled respiration with FCCP
FCCP: carbonylcyanide-4-trifluoromethoxyphenylhydrazone
FSR: fractional synthesis rate
GM: leak respiration with glutamate and malate
GM3: state 3 respiration with glutamate, malate
GMS3: state 3 respiration with glutamate, malate and succinate
HLRE: high-load resistance exercise
MIDA: mass isotopomer distribution analysis
p38 MAPK: p38 mitogen-activated protein kinase
PVDF: polyvinylidene difluoride
QQ-plot: quantile-quantile plot
RCR: respiratory control ratio
RM: repetition maximum
ROS: reactive oxygen species
SD: standard deviation
SDS-PAGE: sodium dodecyl sulfate poly-acrylamide gel electrophoresis
