# Supplementary material for: Skeletal Muscle Mitochondrial Protein Synthesis and Respiration Increase With Low-Load Blood Flow Restricted as Well as High-Load Resistance Training
Source: Front Physiol. 2018 Dec 17;9:1796. doi: 10.3389/fphys.2018.01796 (PMC6304675; doi:10.3389/fphys.2018.01796)
Supplement: Supplementary file 1 [file Table_1.DOCX]

| **SUPPLEMENTARY TABLE 1 \| CORRELATION ANALYSES** | | | | | | |
| --- | --- | --- | --- | --- | --- | --- |
| **Correlation between mitochondrial protein FSR and changes in mitochondrial function and CS activity** | | | | | | |
|  | **ΔGM** | **ΔGM3** | **ΔGMS3** | **Δ4o** | **ΔE** | **ΔCS activity** |
| **All groups** | r = -0.04  P = 0.88 | r = 0.12  P = 0.64 | r = 0.44  P = 0.07 | r = 0.05  P = 0.85 | r = 0.25  P = 0.31 | r = 0.23  P = 0.32 |
| **Training** | r = -0.15  P = 0.63 | r = -0.18  P = 0.58 | r = -0.12  P = 0.71 | r = -0.07  P = 0.82 | r = -0.21  P = 0.51 | r = -0.07  P = 0.80 |
| **HLRE** | r = -0.75  P = 0.06 | r = -0.77  P < 0.05* | r = -0.40  P = 0.38 | r = -0.07  P = 0.88 | r = -0.34  P = 0.45 | r = -0.07  P = 0.88 |
| **BFRRE** | r = -0.12  P = 0.84 | r = -0.44  P = 0.45 | r = -0.35  P = 0.56 | r = 0.01  P = 0.99 | r = -0.67  P = 0.21 | r = 0.32  P = 0.54 |
| **Correlation between mitochondrial protein FSR and post-intervention mitochondrial function and CS activity** | | | | | | |
|  | **GM** | **GM3** | **GMS3** | **4o** | **E** | **CS activity** |
| **All groups** | r = 0.09  P = 0.63 | r = 0.38  P < 0.05* | r = 0.45  P < 0.05* | r = 0.31  P = 0.09 | r = 0.24  P = 0.19 | r = -0.13  P = 0.46 |
| **Training** | r = 0.02  P = 0.92 | r = 0.13  P = 0.57 | r = 0.02  P = 0.92 | r = 0.08  P = 0.74 | r = 0.07  P = 0.76 | r = -0.23  P = 0.30 |
| **HLRE** | r = -0.22  P = 0.52 | r = 0.37  P = 0.27 | r = 0.29  P = 0.39 | r = 0.15  P = 0.66 | r = 0.15  P = 0.66 | r = -0.45  P = 0.14 |
| **BFRRE** | r = 0.31  P = 0.39 | r = -0.16  P = 0.65 | r = -0.20  P = 0.58 | r = 0.02  P = 0.96 | r = -0.03  P = 0.94 | r = -0.10  P = 0.76 |

*Correlation analyses between mitochondrial protein fractional synthesis rate (FSR) and mitochondrial function and CS activity. All groups, all groups pooled; Training, training groups pooled; HLRE, high-load resistance exercise group only; BFRRE, blood flow restricted resistance exercise group only. GM, state 2 respiration; GM3, complex I supported state 3 respiration; GMS3, complex I and II supported state 3 respiration; 4o, state 4 respiration; E, maximal uncoupled respiration; CS activity, citrate synthase activity. * denotes significant correlation.*
